# Psoriasis Google Trends

**DOI:** 10.2196/21709

**Published:** 2021-06-08

**Authors:** Fernando Garcia-Souto, Jose Juan Pereyra-Rodriguez

**Affiliations:** 1 Department of Dermatology Valme University Hospital Sevilla Spain; 2 Department of Dermatology Virgen del Rocio University Hospital Sevilla Spain

**Keywords:** Google Trends, psoriasis, treatment

## Research Letter

In recent years, the internet has become an essential tool where people seek information about health care [1]. The use of the internet as a health resource is increasing rapidly for both patients and health care professionals, playing an important role in the decision-making process [2]. Google Trends is a free and easily accessible web search tool that allows estimating interest in topics at the population level by analyzing all search queries for a specific term in various regions and languages [3].

The aim of this study is to use data from Google Trends to analyze worldwide public interest in psoriasis and its different treatment modalities, and to analyze the possible seasonality of searches. A worldwide search was carried out through Google Trends from 2004 to 2019. A combination of terms related to psoriasis treatments was introduced. Joinpoint regression was performed. Google Trends assigns a relative search volume index to the search terms. Comparison annual relative search volume, annual percentage change, and average annual percentage change (AAPC) were analyzed to assess loss or gain of interest.

Our study reflected an increase interest in secukinumab (AAPC 33.7), ixekizumab (AAPC 23.3), and apremilast (AAPC 21.4). It showed less interest in methotrexate (AAPC –3.6), retinoids (AAPC –9.8), cyclosporine (AAPC –9.8), phototherapy (AAPC –6.3), etanercept (AAPC –14.9), infliximab (AAPC –14), and adalimumab (AAPC –5.8). Seasonality was found in the search term “psoriasis” (Table 1 and Multimedia Appendix 1).

**Table 1:** Trends in the interest of the world population in psoriasis and its treatment modalities.

Range (joint point) and period						Change year			APC^a^ (95% CI)			AAPC^b^ (95% CI)			*P* value	
**Psoriasis (1)**																
	2004-2019				N/A^c^			N/A			0.4 (–0.4 to 1.3)			.30		
	2004-2008				2008			–7.2 (–10.0 to –4.3)			N/A			<.05^d^		
	2008-2019				N/A			3.4 (2.8 to 4.0)			N/A			<.05		
**Psoriasis +^e^ treatment (2)**																
	2004-2019				N/A			N/A			1.6 (–4.2 to 7.7)			.60		
	2004-2007				2007			–3.9 (–18.8 to 13.8)			N/A			.60		
	2007-2010				2010			18 (–12.2 to 58.6)			N/A			.20		
	2010-2019				N/A			–1.6 (–3.2 to 0.1)			N/A			.10		
**Psoriasis + clinical trial (1)**																
	2004-2019				N/A			N/A			–9.5 (–14.1 to –4.6)			<.05		
	2004-2011				2011			–18.8 (–24.4 to –12.9)			N/A			<.05		
	2011-2019				N/A			–0.4 (–9.1 to 9.1)			N/A			.90		
**Classic systemic therapies**																
	**Psoriasis + methotrexate (1)**															
		2004-2019			N/A			N/A			–3.6 (–5.3 to –1.8)			<.05		
		2004-2007			2007			–24.4 (–30.6 to –17.7)			N/A			<.05		
		2007-2019			N/A			2.5 (1.2 to 3.8)			N/A			<.05		
	**Psoriasis + retinoids (0)**															
		2004-2019			N/A			N/A			–9.8 (–13.7 to –5.7)			<.05		
	**Psoriasis + cyclosporine (1)**															
		2004-2019			N/A			N/A			–9.8 (–12.1 to –7.4)			<.05		
		2004-2007			2007			–34.7 (–41.4 to –27.2)			N/A			<.05		
		2007-2019			N/A			–2.2 (–4.5 to 0.2)			N/A			.10		
	**Psoriasis + apremilast (1)**															
		2007-2019			N/A			N/A			21.4 (10.1 to 33.8)			<.05		
		2007-2014			2014			37.1 (15.1 to 63.3)			N/A			<.05		
		2014-2019			N/A			2.3 (–9.6 to 15.9)			N/A			.70		
	**Psoriasis + phototherapy (1)**															
		2004-2019			N/A			N/A			–6.3 (–9.6 to –2.9)			<.05		
		2004-2008			2008			–22.6 (–31.3 to –12.9)			N/A			<.05		
		2008-2019			N/A			0.4 (–3.0 to 3.9)			N/A			.80		
**Biological therapies**																
	**TNF^f^ inhibitors**															
		**Psoriasis + etanercept (0)**														
			2004-2019	N/A			N/A			–14.9 (–18.2 to –11.4)			<.05			
		**Psoriasis + infliximab (1)**														
			2004-2019	N/A			N/A			–14 (–18.7 to –9.0)			<.05			
			2004-2012	2012			–21.6 (–25.4 to –17.5)			N/A			<.05			
			2012-2019	N/A			–4.4 (–15.5 to 8.2)			N/A			.40			
		**Psoriasis + adalimumab (0)**														
			2004-2019	N/A			N/A			–5.8 (–8.3 to –3.3)			<.05			
		**Psoriasis + certolizumab (1)**														
			2007-2019	N/A			N/A			6.5 (–5.3 to 19.8)			.30			
			2007-2013	2013			–10 (–27.4 to 11.6)			N/A			.30			
			2013-2019	N/A			26 (6.0 to 49.8)			N/A			<.05			
	**IL^g^-17 inhibitors**															
		**Psoriasis + secukinumab (1)**														
			2011-2019	N/A			N/A			33.7 (15.0 to 55.4)			<.05			
			2011-2014	2014			112 (22.0 to 268.4)			N/A			<.05			
			2014-2019	N/A			1.4 (–6.2 to 9.6)			N/A			.60			
		**Psoriasis + ixekizumab (1)**														
			2012-2019	N/A			N/A			23.3 (5.8 to 43.8)			<.05			
			2012-2016	2016			60.7 (12.8 to 129.1)			N/A			<.05			
			2016-2019	N/A			–13.3 (–38.2 to 21.5)			N/A			.30			
		**Psoriasis + brodalumab (1)**														
			2012-2019	N/A			N/A			17.2 (–9.0 to 50.8)			.20			
			2012-2014	2014			83.6 (–53.1 to 619.7)			N/A			.30			
			2014-2019	N/A			–2.1 (–17.8 to 16.5)			N/A			.70			
	**IL-23 inhibitors**															
		**Psoriasis + ustekinumab (2)**														
			2007-2019	N/A			N/A			6.1 (–1.3 to 14)			.10			
			2007-2009	2009			102.3 (25.4 to 226.3)			N/A			<.05			
			2009-2012	2012			–27.9 (–40.5 to –12.7)			N/A			<.05			
			2012-2019	N/A			4.1 (1.0 to 7.3)			N/A			<.05			
		**Psoriasis + guselkumab (0)**														
			2013-2019	N/A			N/A			17.7 (–4.7 to 45.4)			.10			
		**Psoriasis + risankizumab (0)**														
			2016-2019	N/A			N/A			33.6 (–43.3 to 214.7)			.30			
		Psoriasis + tildrakizumab			N/A			N/A			N/A			N/A		

^a^APC: annual percentage change.

^b^AAPC: annual average percentage change.

^c^N/A: not applicable.

^d^Exact *P* values not available when *P*<.05.

^e^The “+” sign was not used in the searches. They have only been included in the table to make it easier for the reader.

^f^TNF: tumor necrosis factor.

^g^IL: interleukin.

The results of our study revealed that the overall number of searches for psoriasis decreased between 2004 and 2008 but has steadily increased since then. The general interest in psoriasis treatments decreased between 2004 and 2007, increased considerably until 2010, and since then has decreased slightly. In this study, apremilast and especially secukinumab and ixekizumab have been the treatments that have aroused the most interest. Contrastingly, it reflects a significantly lower interest in methotrexate, retinoids, cyclosporine, phototherapy, etanercept, infliximab, and adalimumab.

A seasonality analysis was performed with the term “psoriasis” to assess whether there is a seasonal variation in interest. During the years 2004 to 2019, there was a regular increase in interest in the period from January to April, which corresponds to the winter and spring months in the northern hemisphere. Likewise, lower interest was frequently recorded in the months of June to September, which correspond to the summer months in the northern hemisphere. Seasonality was not observed in the rest of the variables included in the study. Although the pathogenesis of psoriasis remains unknown, it is well known that certain environmental factors may influence its pathogenesis [4].

In conclusion, our results show current search trends for psoriasis and the various approved systemic treatments based on Google Trend analysis. We consider that the results of our study are useful to identify the search trends of the population on the web. It is essential that public health systems take these data into consideration since searches through the internet give us relevant information about the interest and concerns of the population about their diseases.

## Appendix

Multimedia Appendix 1 Joint point models for the different terms included in the study. RSV: relative search volume index.

## Abbreviations

AAPC: average annual percentage change
