# Supplementary figures and images for: Psoriasis Google Trends
Source: JMIR Dermatol. 2021 Jun 8;4(1):e21709. doi: 10.2196/21709 (PMC10501518; doi:10.2196/21709)

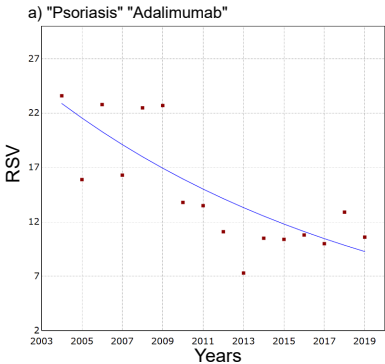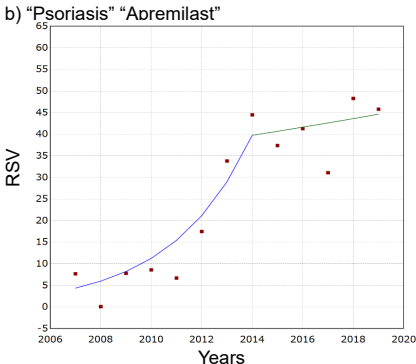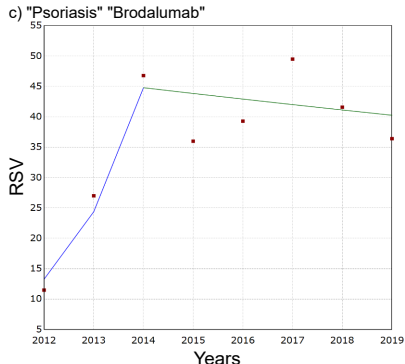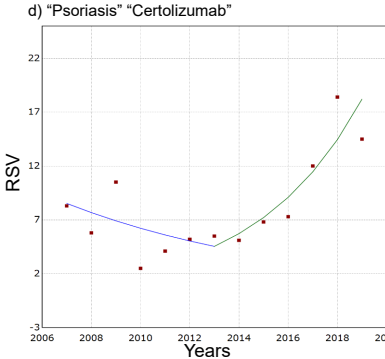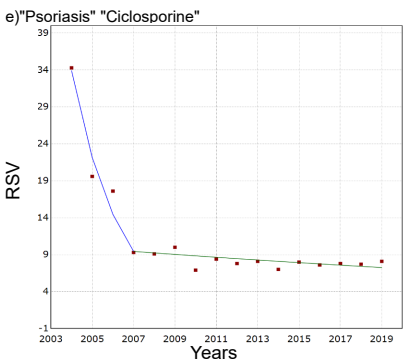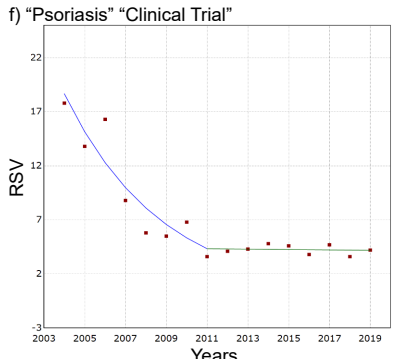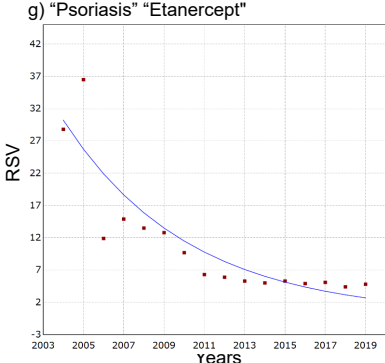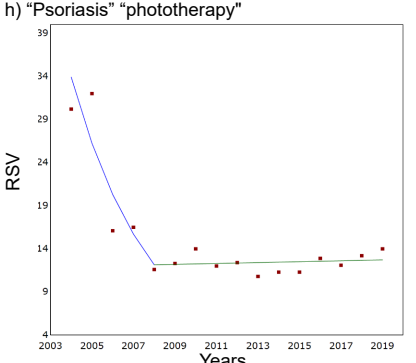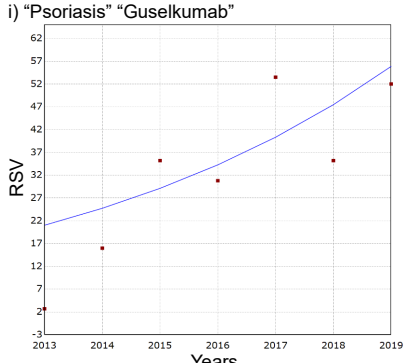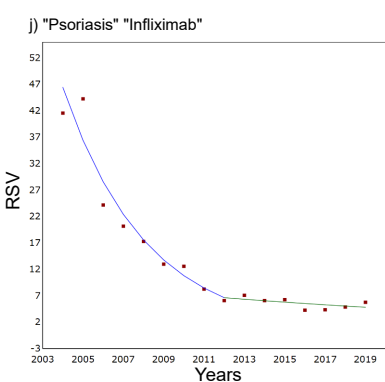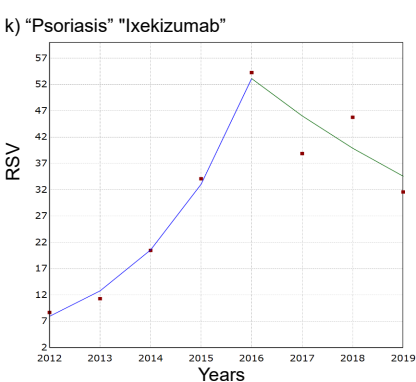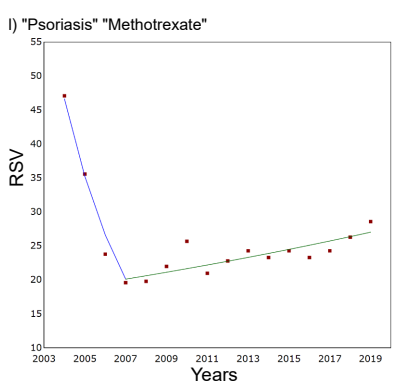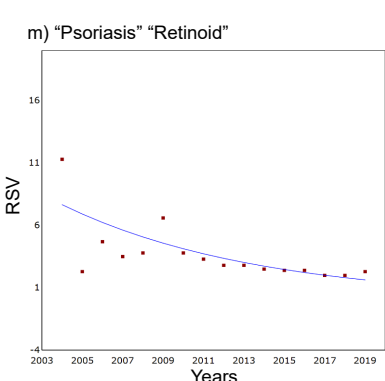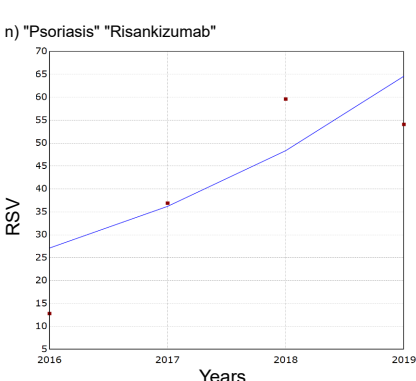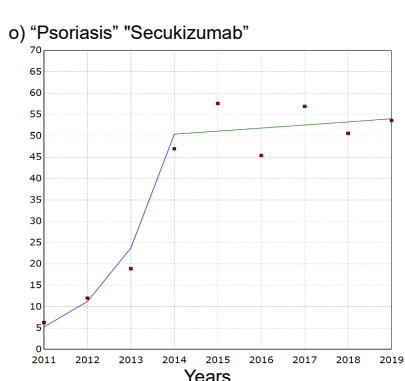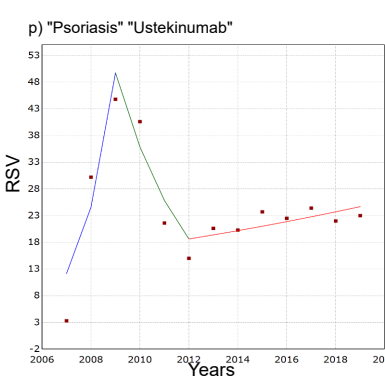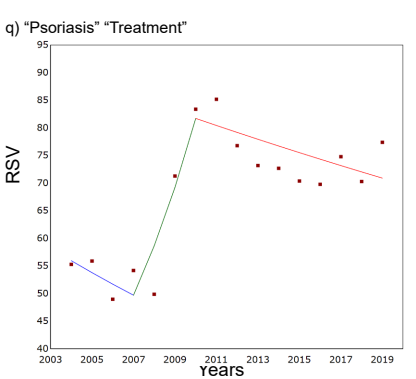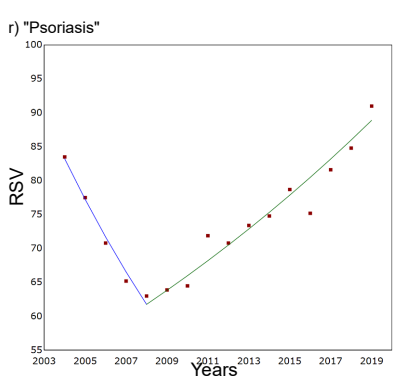

Supplement: Multimedia Appendix 1 [file derma_v4i1e21709_app1.pdf]
